# How do family firms balance economic and non-economic goals: from symbiosis to competition

**DOI:** 10.3389/fpsyg.2025.1538103

**Published:** 2025-06-18

**Authors:** Wei Zhang, Bingde Wu, Ling Chen, Jian-an Zhu, Shihui Chen

**Affiliations:** ^1^School of Management, Zhejiang University of Science and Technology, Hangzhou, China; ^2^School of Economics and Management, Fuzhou University, Fuzhou, China; ^3^School of Management, Zhejiang University, Hangzhou, China; ^4^School of Business, Hangzhou City University, Hangzhou, China; ^5^School of Business, Ningbo University, Ningbo, China

**Keywords:** family firms, non-economic goals, economic goals, socioemotional wealth, family managers

## Abstract

**Introduction:**

The coexistence of non-economic and economic goals is a prominent feature of family firms. However, does the pursuit of non-economic goals necessarily imply that the economic goals should be sacrificed? Our research addresses this question by exploring the symbiotic or competitive relationship between non-economic goals and economic goals in Chinese family firms, and the moderating effect of firm size and firm age.

**Methods:**

Based on 2877 firm-year observations of Chinese listed family firms from year 2009 to 2019, this paper examines the relationship between non-economic goals (measured by family management) and economic goals (measured by firm performance). A panel data fixed-effects regression model was employed for the primary analysis. To further ensure the credibility of our conclusions, we performed several robustness tests, such as utilizing alternative variable measurement and conducting an endogeneity test.

**Results:**

The empirical analysis revealed an inverted U-shaped relationship between family management and firm performance, where the extent to which non-economic goals are positively related to economic goals up to a point, after the turning point it becomes negative, which shows the trend from symbiosis to competition. Furthermore, as firm age increases and firm size expands, the inverted U-shaped curve flattens, and the turning point shifts to the right.

**Discussion:**

Employing a willingness and ability perspective, this research contributes to the socioemotional wealth (SEW) framework by offering insights into the dynamic interplay between economic and non-economic goals in Chinese family firms. Moreover, by examining Chinese family firms influenced by Confucian values, our study highlights the importance of cultural context for generalizability, while simultaneously enriching SEW discourse and fostering avenues for cross-regional comparative analysis.

## 1 Introduction

Entrepreneurs often face the dilemma of balancing multiple goals in daily business practices. However, in academic research, the majority of economic and management studies typically assume the maximization of economic goals for theory construction (Vazquez and Rocha, 2018), as seen in agency theory (Jensen and Meckling, 1976) and resource-based view (Teece et al., 1997). Although stewardship theory (Davis et al., 1997) and stakeholder theory (Parmar et al., 2010) emphasize that different individuals have different triggers and goals, they do not explain how these goals interact. This gap remained until the emergence of socioemotional wealth (SEW) (Gómez-Mejia et al., 2023). SEW is defined as the nonfinancial value embedded in family firms, highlighting their tendency to prioritize non-economic goals over economic ones in decision-making, thereby differentiating family firms from nonfamily firms (Gomez-Mejia et al., 2007; Gomez-Mejia et al., 2011; Gomez-Mejia et al., 2018; Gómez-Mejia et al., 2023). It highlights the non-economic nature of family firms and has gradually become the mainstream paradigm in family firm research (Swab et al., 2020).

The coexistence of non-economic and economic goals is a prominent feature of family firms (Dou et al., 2022). However, does the pursuit of non-economic goals necessarily imply that the economic goals should be sacrificed? Due to the popularity of SEW, the primacy of non-economic goals and the perceived antagonism between the economic and non-economic goals are increasingly being taken for granted (Vazquez and Rocha, 2018). Considering the VUCA (volatile, uncertain, complex, ambiguous) environment, and heterogeneity of family firms (Daspit et al., 2021), there is room for adjustment and development in SEW’s discourse on the multiple goal relationship (Gómez-Mejia et al., 2023; Gomez-Mejia et al., 2024). Moreover, the pursuit of multiple goals in Chinese family firms is deeply embedded in diverse cultural context (Koellner and Roth, 2024), such as “family relationship” (guanxi), familial bonds (Su et al., 2023) and nepotism, positioning the family not merely as an economic unit but as a cornerstone of non-economic driver. These cultural imperatives fundamentally shape the prioritization of non-economic goals, which operate symbiotically with economic imperatives.

Enterprises typically pursue satisfactory rather than optimal goals, a dynamic further complicated by the cultural context and developmental stages of family firms operating in VUCA environment. Our research integrates these insights into the SEW framework by exploring the symbiotic or competitive relationship between non-economic goals (e.g., family management) and economic goals (e.g., firm performance) in Chinese family firms.

Non-economic goals constitute a multi-dimensional concept, among which maintaining family control is the primary dimension and serves as the foundation for achieving other non-economic goals (Berrone et al., 2012; Dou et al., 2020). According to upper echelons theory, top management teams significantly influence a firm’s strategy, behavior, and performance (Hambrick, 2007). Furthermore, the identity of managers, whether family or non-family, profoundly affects firm performance (Zhang et al., 2021; Fang et al., 2022). Therefore, family members serving as managers become the most important manifestation of maintaining family control. In this study, we use family members serving as managers to represent non-economic goals, while firm performance represents economic goals.

We adopt a goal-goal perspective to examine the relationship between the two goals, yielding distinct research results compared to the traditional means-goal perspective. According to the means-goal perspective, the relationship between family involvement and firm performance is widely debated. When firm performance is viewed as the goal pursued by family firms, and family involvement is considered the means to achieve this goal, scholars have identified various results (Patel and Cooper, 2014). Shifting from the means-goal perspective to a goal-goal perspective offers a more compelling explanation. A single goal can be approached with maximization logic, while multiple goals involve subjective weighting and can only be addressed using satisfying or optimization algorithms, which rely on the decision-maker’s subjective value standards (Meng et al., 2023). According to this logic, there may not be a simple optimal relationship between family management and firm performance. Instead, there may be trade-offs from a goal-goal perspective, reflecting differences in decision-makers’ value standards. Adopting a willingness-ability perspective, we propose a model demonstrating a shift from “symbiosis” to “competition” between the proportion of family management (as a non-economic goal) and firm performance (as an economic goal). Symbiosis occurs when economic goals and non-economic goals increase together, reflecting a synergistic alignment, and competition emerges when further increases in non-economic goals lead to a decline in economic goals, signaling trade-offs, forming a non-differentiated inverted U-shaped curve for the dominant family.

The relationship between economic and non-economic goals also shifts with a firm’s internal and external environment. As firms age and grow, they develop more standardized norms and practices, leading to standard organizational roles and control systems. These standardized practices enhance organizational credibility and establish clear accountability (Hannan and Freeman, 1984). Larger and older companies rely more on systematic processes than on individual managers’ capabilities, mitigating deficiencies in less capable family managers. Moreover, family managers possess sufficient authority to bypass internal processes and regulations, thereby reducing the impact of structural inertia, which allows the organization to combine the reliability brought by standardization with the flexibility afforded by discretionary power (Ahmad et al., 2021). Consequently, changes in firm age and size will affect the willingness and ability of family managers, consequently influencing the “symbiosis” and “competition” relationships between the economic and non-economic goals of family firms.

Using data from Chinese listed family firms from 2009 to 2019, we selected family management to represent non-economic goals and firm performance to represent economic goals, and explores the relationship between these two types of goals in family firms in long-term stable conditions. The study examines the symbiosis and competition relationships between these goals, as well as the moderating effects of firm size and firm age. This research provides new insights into the field of corporate goals and socio-emotional wealth, and clarifies misconceptions regarding the professionalization of family firms.

## 2 Theoretical background

Family firms simultaneously pursue economic and family-centered non-economic goals (Dou et al., 2022). While economic goals, such as profitability and growth, remain central to the survival and competitiveness of family firms. Non-economic goals, particularly those centered on family well-being and SEW, play a pivotal role in shaping their strategic decisions (Chua et al., 2018). Some researches highlight the diversity of family firms’ goals, often classified into binary categories such as economic versus non-economic or financial versus non-financial (Vazquez and Rocha, 2018). While mainstream theories such as agency theory suggests that the goal pursuits of family firms and non-family firms do not exhibit significant differences and primarily diverge in the governance structure (Saleem and Graves, 2024). Similarly, the resource-based view emphasizes that family firms’ unique resource advantages can generate greater economic benefits, with these resources serving as tools for realizing economic benefits (Habbershon et al., 2003). Stewardship theory changes the foundational assumptions of human nature but retains the basic premise that a firm’s stakeholders primarily seek economic benefits (Fox and Hamilton, 1994). Stakeholder theory, while acknowledging diverse goal pursuits among stakeholders lacks a practical framework for analyzing these differences (Donaldson and Preston, 1995). Scholars have gradually recognized that family and non-family firms differ not only in governance structures but also in non-economic goals that influence corporate behavior (Chrisman et al., 2012).

Gomez-Mejia et al. (2007) developed the SEW framework, systematically explaining non-economic goals and distinguishing family firms from their non-family counterparts. Furthermore, Berrone et al. (2012) developed a multi dimensional SEW model identifies five non-economic values (FIBER) that family owners prioritize: (a) Family control and influence, (b) Family members’ identification with the firm, (c) Binding social ties, (d) Emotional attachment and (e) Renewal of family bonds to the firm through dynastic succession. This model represents a significant advancement in understanding the intrinsic structural composition of SEW (Schulze and Kellermanns, 2015). Furthermore, using FIBER as an overarching framework, Gomez-Mejia et al. (2024) distinguish between SEW-intensive—where firms give high priority to the FIBER dimensions—and SEW-sensitive—where family owners are more willing and capable of adapting their SEW endowment to respond to external factors. In the Chinese context, family firms also exemplify both traits: their SEW-intensive nature is driven by cultural imperatives such as “guanxi,” familial bonds, and nepotism (Su et al., 2023), while their SEW sensitivity emerges from navigating a VUCA environment shaped by rapid economic and institutional transitions (Koellner and Roth, 2024). This dual characters enrich our understanding of how Chinese family firms navigate the interplay between economic and non-economic goals. Leveraging its strength in explaining non-financial motivations of family firms, SEW exerted extensive influence across organizational and management studies, including corporate governance (Mahto et al., 2023), internationalization (Hafner and Pidun, 2022), innovation and entrepreneurship (Hu et al., 2023), and social ethics (Kabbach de Castro et al., 2017). This has led to the assumption that the pursuit of SEW often comes at the expense of economic interests, reinforcing the notion of a trade-off between economic and non-economic goals (Vazquez and Rocha, 2018). However, considering a dynamic further complicated by the cultural context and developmental stages of family firms operating in China, the relationship between economic and non-economic goals becomes more nuanced, leaving room for further adjustment and development.

Examining the relationship between family management and firm performance through a goal-goal perspective offers a distinct analytical framework compared to the means-goal perspective. Existing research, which treats SEW as an end goal and family control as a means to achieve it, has yielded inconsistent findings. Studies utilizing North American samples have identified a positive correlation between the proportion of professional managers and firm performance (Fang et al., 2022), suggesting an inverse relationship between family management and firm performance. Conversely, research based on Italian samples has demonstrated an inverted U-shaped relationship between the presence of family managers and firm performance (Sciascia and Mazzola, 2008). Against this backdrop, and in light of the extensive discourse on non-economic goals in family firms (Gomez-Mejia et al., 2011), a critical question emerges: Given that family control can either facilitate or hinder the attainment of economic goals, how should family firms strategically balance or coordinate the degree of family management versus professionalization? To address this question, this paper proposes a goal-goal perspective to systematically explore the interplay between the pursuit of non-economic goals and the achievement of economic goals in family firms.

## 3 Hypothesis

### 3.1 The relationship between non-economic goals and economic goals

We adopt a willingness-ability perspective to analyze the relationship between non-economic goals (family management) and economic goals (firm performance). Family managers possess the willingness to pursue specific goals, which is shaped by their values, beliefs, and family ties. However, their ability to achieve these goals is constrained by factors such as resource availability, market conditions, and managerial expertise. By examining both willingness and ability, we can gain a more nuanced understanding of how family managers navigate the trade-offs between non-economic and economic goals and ultimately influence firm performance.

The attainment of economic goals is essential for firm survival, and family managers possess the willingness to achieve them. First, the tradition of Chinese culture emphasizes familial loyalty, collective responsibility, and long-term orientation (Frerich et al., 2024), which closely aligning firm’s survival and development with their own interests. These cultural norms foster a deep sense of commitment and trust (Hennicks et al., 2024) to the firm’s success, as family managers also exhibit higher organizational loyalty and commitment (Luo and Chung, 2013), which strengthens their dedication to realizing both economic and non-economic goals. Second, personal connections and “guanxi” based on blood and marriage ties provide family managers with a natural foundation of trust. The long periods of living and working together help family managers develop shared values and understanding (Bettinelli et al., 2022), maintain high visibility in their work, and ensure relatively transparent communication channels, thereby reinforcing their willingness to drive the firm toward its goals. This reduces opportunistic tendencies and lowers agency costs (Fama and Jensen, 1983; Karra et al., 2006), enabling them to focus more on achieving economic goals. In the context of China, which could be considered a highly SEW-intensive environment due to the strong emphasis on family ties and long-term relationships (Gomez-Mejia et al., 2024), the willingness to prioritize goals may be particularly pronounced.

From the willingness perspective, all else being equal, an increase in the proportion of family managers initially benefits the entire top management team by reducing agency costs, primarily due to diminished information asymmetry (Chrisman et al., 2004). However, as this proportion continues to rise, the team may begin to experience escalating agency costs driven by family altruism. Family owners’ altruism may manifest as tolerance and generosity towards family managers, potentially leading to shirking and free-riding behaviors (Schulze et al., 2003), thereby increasing agency costs (Chrisman et al., 2004). Thus, considering the overall agency costs, while the willingness of family managers to achieve the firm’s economic goals grows with their increasing proportion, it follows a pattern of diminishing marginal utility.

From the ability perspective, family managers may face ability disadvantages. In the early development stage of a family firm, maintaining family control generates “familiness,” which enhances the firm’s performance by leveraging capabilities and resource advantages (Wu et al., 2024). However, as the proportion of family managers increases, the average ability level within the entire TMTs tends to decline, which is attributed to the higher degree of homogeneity in their acquired knowledge and skills, as well as significant overlap in their social capital (Gómez-Mejia et al., 2023). The homogeneity and overlap imply that the ability of the whole TMT do not increase proportionally with the increase in the proportion of family managers. Moreover, the continuous involvement of family managers often favors kinship over meritocracy, preventing the firm from benefiting from the diverse capabilities and resources brought by professional managers (Chua et al., 2009). Additionally, nepotism, which leads to position protection by the owner, may result in the TMT being filled with incompetent family managers (Schulze et al., 2003). This trend is exacerbated by the increasing presence of underqualified family managers (Stewart and Hitt, 2012), leading to a decline in the firms’ competence.

The achievement of economic goals can be seen as a combined effect of ability and willingness of family managers (De massis et al., 2014). When the proportion of family managers is low, they possess the willingness to achieve economic goals and have not yet demonstrated significant ability disadvantages, and may even exhibit slight ability advantages. At this stage, the joint effect of increasing willingness and the maintenance of ability leads to a positive effect on economic performance. As the proportion of family managers increases, the diminishing effect of willingness advantages and the increasing effect of ability disadvantages result in a positive net effect on firm performance that continues to grow but at a diminishing marginal rate. At this stage, family management demonstrates a mutually “symbiotic” relationship with the firms’ economic goals. However, constrained by the law of diminishing marginal utility, as the proportion of family managers increases, firm performance will gradually reach a point where the marginal benefit becomes zero, known as the turning point. Subsequently, due to the continuous involvement of family managers, their ability to achieve economic goals continues to decline, while the increasing rate of willingness also diminishes. The combined effect of willingness and ability on firm performance begins to decline, accelerating over time. At this stage, family management shifts from enhancing the firm’s economic goals to eroding them, demonstrating a “competition” relationship. This analysis indicates that the relationship between non-economic and economic goals shifts from symbiotic to competitive. Based on these theoretical arguments, the following hypothesis is proposed:

*Hypothesis 1: There will be an inverted U-shaped relationship between family management and firm performance, that is to say, where the extent to which non-economic goals are positively related to economic goals up to a point, after which it becomes negative.*

### 3.2 The moderating effects of firm characteristics

Characteristics such as firm size and firm age influence a firm’s survival (Bruderl and Schussler, 1990), innovation (Gimenez-Fernandez et al., 2020), and other strategic decisions. In line with the findings of Gomez-Mejia et al. (2024), larger and older firms in SEW-sensitive contexts are better positioned to preserve socioemotional wealth while pursuing economic goals, as they can allocate resources more effectively and absorb external shocks, and they are better equipped to navigate VUCA environments. As firms grow in size and age, formal and informal norms mature, organizational practices stabilize, and the resource endowment of the firm, along with the emotional endowment of the controlling family, become key elements in determining the firm’s strategy. Consequently, the “symbiosis” and “competition” relationship between economic and non-economic goals of family firms are influenced by these characteristics.

#### 3.2.1 The moderating effect of firm size

Firms with larger size are able to amplify the willingness advantage and mitigate the ability disadvantage of family managers through systemic forces. The willingness advantage is further reinforced in larger firms. First, compared to smaller firms, larger firms are more likely to benefit from economies of scale and scope. Their complementary assets and capabilities, higher risk tolerance, and stronger market positions all contribute to improving the internal efficiency of resource conversion. The scale effect amplifies the willingness advantage of family managers, enabling them to generate higher economic returns from the same level of resource endowment. Second, larger family firms are more incentivized to establish standardized operational processes and governance structures, as well as to offer formal and effective compensation incentives to managers (Carlson et al., 2006). In this context, the altruistic tendencies of the owners are naturally diminished, thereby reducing the costs associated with position mismatches due to altruism (Schulze et al., 2003). This decline in agency costs further strengthens the willingness of family managers to dedicate their efforts toward achieving the firm’s goals.

Larger firms can also partially compensate for the shortcomings of family managers’ insufficient abilities. First, as firms grow in size, they have more resource to establish modern governance and management systems, as well as industry norms (Carlson et al., 2006). These firms can also provide formal training for employees and managers, thereby improving the competence of the entire top management teams, including family managers. Second, larger firms can more easily achieve economies of scale, enhance production efficiency, and implement more detailed labor divisions. This allows the organization ability to substitute for individual ability, partially mitigating the deficiencies of family managers. Since the impact of family managers’ willingness and ability on firm performance changes with firm size, the slope and turning point of the inverted U-shaped curve will also change accordingly.

Larger firms are more conducive to leveraging the willingness advantage of family managers while mitigating their ability disadvantages. Therefore, in larger firms, as the proportion of family managers increases, the combined effect of the increased willingness advantage and the mitigated ability disadvantage leads to a slower approach to the turning point, resulting in a flatter inverted U-shaped curve. Additionally, larger firms can accommodate more family managers to achieve their economic goals. Consequently, the point at which the marginal net effect on firm performance becomes zero shifts to the right, indicating a rightward shift in the turning point of the inverted U-shaped curve. Taken together, these theoretical arguments yield the following hypothesis:

*Hypothesis 2: Firm size moderates the inverted U-shaped relationship between family management and firm performance, such that at larger firms, the inverted U-shaped becomes flatter, and the turning point shifts to the right.*

#### 3.2.2 The moderating effect of firm age

Firms with older age typically accumulate extensive management experience and organizational routines, resulting in smoother communication mechanisms. For family firms, as the firm ages, the intention for intergenerational succession and maintaining family control gradually emerges (Chua et al., 2004). Thus, firm age can be considered a dynamic representation of the firm’s development stage, allowing us to examine changes in the relationship between economic and non-economic goals over different development stage.

In older firms, the willingness advantages of family managers are reinforced for several reasons. First, family managers develop a strong sense of “psychological ownership” due to their long-term involvement with the firm (Lee et al., 2019). Under the influence of stronger emotional endowments, the same proportion of family managers demonstrate a greater willingness to achieve the firm’s economic goals (Zellweger et al., 2011). Second, in older firms, family managers establish effective formal or informal communication channels through long-term operations and continuous collaboration. Their “tacit knowledge” facilitates smoother communication, effectively reducing information asymmetry and enhancing self-monitoring capabilities (Karra et al., 2006). Consequently, the willingness of family managers to invest their efforts can yield greater competitive advantage.

Firm age also impacts the ability endowments of family managers in several ways. First, the management experience and practices accumulated over time in older firms make daily operations more dependent on inertia, which inadvertently mitigates or eliminates the potential impact of family managers’ ability disadvantages. Second, in long-established family firms, family managers contribute to the development of tacit knowledge suitable for the firm through years of learning, integration, and accumulated experience (Miller et al., 2011). Over time, family managers build corporate reputation, market share, and customer resource, which external professional managers cannot easily replicate. Furthermore, this tacit knowledge further mitigates the negative impact of family managers’ ability deficiencies.

Since the impact of family managers’ willingness and ability on firm performance varies with firm age, the slope and turning point of the inverted U-shaped curve change accordingly. Older firms are more conducive to family managers leveraging their willingness advantage and mitigating ability deficiencies. Therefore, in older firms, as the involvement family managers increases, the combined effect of increased willingness and decreased ability deficiencies results in a flatter inverted U-shaped curve. Additionally, older firms can accommodate more family managers to achieve economic goals, leading to a rightward shift in the turning point. Thus, considering the variations in the impact of family managers’ willingness and ability endowments on firm performance across different firm age, we propose the following hypothesis:

*Hypothesis 3: Firm age moderates the inverted U-shaped relationship between family management and firm performance, such that at larger firms, the inverted U-shaped curve becomes flatter, and the turning point shifts to the right.*

## 4 Methodology

### 4.1 Data source

The empirical data used in this study were derived from Chinese family firms listed on the Shenzhen and Shanghai stock exchanges between 2009 and 2019. The 2009–2019 time period was selected for the following reasons: This period represents a relatively stable economic phase in China’s development, free from the extreme disruptions caused by the COVID-19 pandemic that began in 2020. By focusing on pre-pandemic data, we can examine the fundamental relationships of family firms’ goal setting strategies under a long-term normal condition. To avoid the interference of this extreme exogenous event on our research findings and to ensure the robustness and generalizability of our conclusions, we utilized the data range to the pre-pandemic period of 2009–2019.

The primary data source was the China Stock Market and Accounting Research (CSMAR) database. Additionally, we collected data from the firms’ annual reports. Information on managers’ family or non-family identity, family management, ROE (return on equity), firm size and firm age, among other variables, was obtained from the CSMAR database. The sample firms are all family firms. We define family firms as follows: First, as recommended by La Porta et al. (1999), we adopted a threshold of 20% family ownership for firm selection. Second, it required at least one family member, whether related by blood or marriage, to serve as a director, shareholder, or manager (Anderson and Reeb, 2003; Chua et al., 1999). To ensure data reliability, we excluded firms in the financial services industry, as their unique financial statement structures and typically high debt ratios lead to biased regression results.

However, variables such as managers’ baseline information like education background, age, etc., could not be obtained directly from the database, so we manually distinguished and calculated these variables. The process was as follows: first, we downloaded managers’ resumes from the CSMAR database, then searched their names online to determine if they had a familial relationship with the holding family. If such a relationship existed, we coded them as family managers, otherwise, they were coded as non-family managers. Second, we collected information on these managers’ age, education, and whether family members served as CEOs or board directors.

Our dataset is an unbalanced panel for several reasons. First, we excluded samples with incomplete information disclosure to prevent missing values. Second, we omitted data for firms that were sold, went bankrupt, or experienced a change in control during the sample period, though we retained data for these firms prior to these events. Consequently, our unbalanced panel comprises 255 firms, representing 2,877 firm-year observations.

### 4.2 Variable measurement

#### 4.2.1 Dependent variable

Return on equity (ROE) of the sample firms is used to represent firm performance, reflecting economic goals. Additionally, since the impact of family managers on financial performance may not be immediately apparent but generally manifests in the following year, this paper utilizes the average ROE of one-year lag (year _t + 1_) and two-year lag (year _t + 2_) measure firm performance in year t.

#### 4.2.2 Independent variable

Following prior literature, we operationalized family management as the proportion of family managers relative to all managers employed in the firm (Block, 2010), which is also the percentage of a firm’s managers who were also family members (Sánchez-Marín et al., 2020).

#### 4.2.3 Moderating variables

Following the research of Fang et al. (2016) and Skorodziyevskiy et al. (2022), this paper incorporates firm size and firm age as moderating variables. Firm size is measured by the logarithm of the operating revenue in the current fiscal year, while firm age is measured by the number of years since the firm was established.

#### 4.2.4 Control variables

In alignment with the previous study of Jaskiewicz et al. (2017), our analysis incorporates a comprehensive set of control variables that are potential determinants of firm performance, systematically categorized into firm characteristics, managerial attributes, corporate governance characteristics and financial attributes. For firm characteristics, we included a variable indicating whether a firm qualifies as a high-tech enterprise, as high-tech industries often exhibit greater performance volatility due to rapid technological advancements and shorter product life cycles. Such volatility can obscure the effects of family management and other variables on firm performance. For managerial attributes, the traits of family managers may influence firm performance (Fredrickson et al., 2010). We controlled for the average age and education level of family managers. Additionally, for corporate governance characters, we controlled for CEO duality, coded as “1” when the CEO also holds the position of chairman of the board. We also controlled for intergenerational succession, as the involvement of second-generation can also influence the firm’s goal setting. For financial attributes, we controlled for the debt-to-asset ratio and beta, which reflects a firm’s financial leverage and debt repayment ability. We also controlled for equity concentration, which reflects the distribution of shareholders’ ownership stakes. High equity concentration typically shows that a few major shareholders have significant control over the firm, directly impacting its decision-making process and governance structure. The variable description is listed in Table 1.

**Table 1:** Variable definitions.

Category	Variable name	Symbol	Measurement
Independent variable	Family management	Family management	The ratio of family managers in TMTs
Dependent variable	Firm performance	Performance	The average ROE (Return on Equity) of year t + 1 and year t + 2.
Moderators	Firm size	Firm size	The natural logarithm of the operating revenue in the current fiscal year.
	Firm age	Firm age	The number of years since the family firm was established.
Control variables	High-tech enterprise	High tech	A binary variable, if a firm is designated as a high-tech enterprise is coded as 1, otherwise 0.
	Independent directors	Independent	The percentage of independent directors in the board of directors.
	CEO duality	Duality	A binary variable, if the CEO also served as the chairman of the board of directors are coded as 1, otherwise 0.
	Intergenerational succession	Succession	If the second generation involved in the firm are coded 1, otherwise 0.
	Debt-to-Asset Ratio	Asset	Debt/Asset
	Beta	Beta	An indicator measures the volatility of a stock relative to the overall market.
	Equity Concentration	Equity	Sum of the shareholding ratios of the 2nd to 5th largest shareholders / Shareholding ratio of the largest shareholder.
	Family managers’ age	FM Age	The natural logarithm of average nonfamily managers’ age.
	Family managers’ education background	FM Edu	1 = Middle school; 2 = Junior college; 3 = Bachelor degree; 4 = Master degree; 5 = PhD.
	Year	Year	Dummy variables, the sample spans from 2009 to 2019 (11 years).
	Industry	Industry	Dummy variables: According to the 2012 industry classification by the China Securities Regulatory Commission (CSRC), the industries covered in the sample are divided into 17 categories.

### 4.3 Data analysis

#### 4.3.1 Descriptive statistics and correlation analysis

Table 2 presents the descriptive statistics and correlation coefficients of the variables. The average ROE of the sample firms is 0.055, with a standard deviation of 0.292, indicating significant differences in firm performance. The average proportion of family managers is 0.169. Regarding firm characteristics, the average firm size and firm age are 21.35 and 2.828. The average age and education level of family managers are 46.02 and 3.104, respectively, which means the average family managers’ age is approximately 46.02 years, and the average education level corresponds to an undergraduate degree.

**Table 2:** Correlation analysis.

	Mean	Std. dev.	1	2	3	4	5	6	7
1. Performance	0.055	0.292	1.000						
2. Family Management	0.169	0.166	−0.003	1.000					
3. Firm Size	21.350	1.222	0.029	−0.135***	1.000				
4. Firm Age	2.828	0.418	−0.037**	−0.099***	0.177***	1.000			
5. High Tech	0.651	0.477	−0.016	0.078***	−0.071***	−0.066***	1.000		
6. Independent	0.374	0.053	−0.022	0.076***	−0.078***	0.061***	0.0280	1.000	
7. Duality	0.358	0.479	−0.040**	0.275***	−0.115***	−0.020	0.035*	0.196***	1.000
8. Succession	0.452	0.498	−0.001	0.155***	0.059***	0.113***	−0.096***	−0.133***	−0.137***
9. Asset	0.682	1.330	0.006	0.013	−0.133***	−0.059***	−0.014	−0.013	−0.016
10. Beta	1.093	0.260	−0.056***	−0.020	−0.071***	0.095***	0.152***	0.012	0.041**
11. Equity	0.537	0.201	0.069***	−0.019	−0.165***	−0.083***	−0.169***	0.007	−0.009
12. FM Age	46.016	3.671	0.001	−0.124***	0.183***	0.195***	−0.0250	0.044**	0.046**
13. FM Edu	3.104	0.545	−0.024	−0.053***	0.097***	0.060***	0.042**	0.048**	0.014

	8	9	10	11	12	13
8. Succession	1.000					
9. Asset	0.001	1.000				
10. Beta	−0.027	−0.039**	1.000			
11. Equity	0.004	0.043**	−0.074***	1.000		
12. FM Age	0.013	−0.027	0.050***	−0.217***	1.000	
13. FM Edu	−0.060***	0.001	0.029	−0.137***	−0.034*	1.000

*p < 0.01, **p < 0.05, ***p < 0.001.

Table 2 also reports the correlation coefficients of the variables. The distribution of sample variables is within a reasonable range and will not be elaborated further here. The correlation coefficients between variables are all below 0.5, indicating no serious multicollinearity problem. Additionally, a Variance Inflation Factor (VIF) test was conducted, showing VIF values for each variable ranging from 1.01 to 1.16, further confirming that multicollinearity is not a significant concern in our research.

#### 4.3.2 Methodology and results

In this study, STATA 15.0 was used for data processing to test the hypothesis. To ensure model consistency and validity, the data were processed as follows: First, continuous variables involved in interaction terms, were centered to avoid the influence of multicollinearity. Second, before conducting the regression tests, a Hausman test was performed. Based on the Hausman test results, the fixed effects regression model for panel data was deemed appropriate for this study, as it also helps to address endogeneity issues.

The regression results were presented in Table 3. First, Model 1 is the baseline model, including only control variables. Model 2 tests the relationship between family management and firm performance by adding both the first-order term (Family Management) and squared term (Family Management^2^) of the independent variable. The results show that the squared term of family management is significantly negative (*β* = −0.320, *p* < 0.05), while the first-order term is significantly positive (*β* = 0.310, *p* < 0.01). This indicates that the relationship between family management and firm performance exhibits an inverted U-shape, meaning that as the ratio of family managers increases, firm performance first rises and then falls. And around the turning point, family firms can reach the optimal performance level. Thus, Hypothesis 1 is supported.

**Table 3:** Regression results.

Variables	Model 1	Model 2	Model 3	Model 4	Model 5
	Performance	Performance	Performance	Performance	Performance
Family Management		0.310^***^	0.273^***^	0.297^***^	0.256^**^		[0.097]	[0.100]	[0.097]	[0.100]
	Family Management^2^		−0.320^**^	−0.243	−0.305^**^	−0.217
		[0.141]	[0.152]	[0.141]	[0.153]
Family Management × Firm Size			−0.103^**^		−0.092^**^
			[0.042]		[0.042]
Family Management^2^ × Firm Size			0.239^**^		0.242^**^
			[0.108]		[0.111]
Family Management × Firm Age				−0.500^***^	−0.493^***^
				[0.122]	[0.123]
Family Management^2^ × Firm Age				0.692^*^	0.799^**^
				[0.394]	[0.407]
Firm Age	0.009	0.006	0.006	0.010	0.007
	[0.025]	[0.025]	[0.025]	[0.026]	[0.026]
Firm Size	−0.006	−0.006	−0.017	−0.003	−0.014
	[0.010]	[0.010]	[0.011]	[0.010]	[0.011]
High Tech	0.009	0.008	0.009	0.004	0.005
	[0.022]	[0.022]	[0.022]	[0.022]	[0.022]
Independent	−0.214	−0.220	−0.248^*^	−0.267^*^	−0.291^**^
	[0.136]	[0.136]	[0.136]	[0.136]	[0.136]
Duality	−0.015	−0.031^**^	−0.032^**^	−0.030^**^	−0.031^**^
	[0.014]	[0.015]	[0.015]	[0.015]	[0.015]
Succession	−0.008	−0.013	−0.014	−0.010	−0.011
	[0.018]	[0.018]	[0.018]	[0.018]	[0.018]
Asset	−0.000	0.000	0.001	0.001	0.001
	[0.004]	[0.004]	[0.004]	[0.004]	[0.004]
Beta	−0.024	−0.027	−0.029	−0.024	−0.026
	[0.020]	[0.020]	[0.020]	[0.020]	[0.020]
Equity	0.100	0.101	0.111^*^	0.095	0.104
	[0.063]	[0.063]	[0.063]	[0.063]	[0.063]
FM Age	0.000	0.001	0.000	0.001	0.001
	[0.002]	[0.002]	[0.002]	[0.002]	[0.002]
FM Edu	−0.000	0.000	0.000	−0.001	−0.001
	[0.016]	[0.015]	[0.015]	[0.015]	[0.015]
Year	Yes	Yes	Yes	Yes	Yes
Industry	Yes	Yes	Yes	Yes	Yes
_cons	0.115	0.086	0.337	0.031	0.279
	[0.251]	[0.251]	[0.270]	[0.251]	[0.271]
adj. *R*^2^	0.181	0.185	0.187	0.190	0.192
*N*	2,877	2,877	2,877	2,877	2,877

*p < 0.1; **p < 0.05; and ***p < 0.01. Standard errors in brackets.

**Figure 1:**
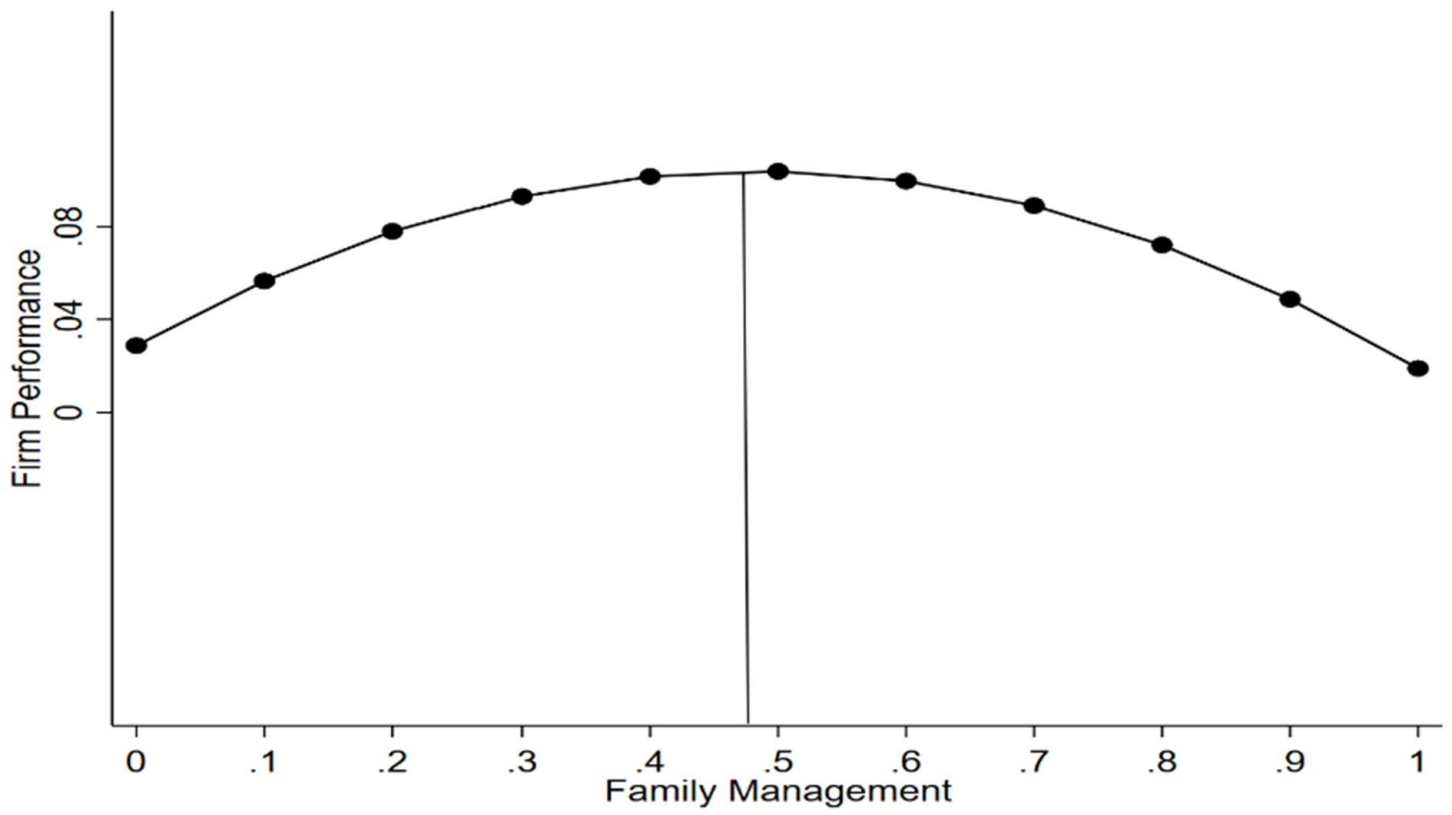
The inverted U-shaped relationship between family management and firm performance.

To ensure the correct interpretation of the results, the significance of the inverted U-shaped relationship was assessed following the method outlined by Lind and Mehlum (2010) (see Table 4). To further verify this relationship, we followed the approach of Haans et al. (2016) and Lind and Mehlum (2010) and conducted the following tests. First, we estimated the turning point of family management and calculated confidence intervals using the Delta method (Fernhaber and Patel, 2012). The turning point of the inverted U-shaped curve between family management and firm performance is 0.485. Additionally, the 95% confidence intervals for the Delta method (0.269, 0.701) indicates that the family management values fall within the data limits. Second, we calculated the slope on both sides of the turning point. The results show that the slope on the left side (X_L_) of the inverted U-shaped curve is significantly positive (Slope_L_ = 0.310, *p* < 0.001), and the slope on the right side (X_R_) of the curve is significantly negative (Slope_R_ = −0.330, *p* < 0.1). Third, following Fernhaber and Patel (2012), we conducted a joint significance test of the direct and squared terms of family management according to the method of Sasabuchi (1980). These validations confirm the existence of the inverted U-shaped relationship between family management and firm performance (see Figure 1).

**Table 4:** Additional test of an inversely U-shaped relationship.

Method	Results
Estimated turning point	0.485
95% confidence interval—Delta method	(0.269, 0.701)
Slope	Slope_L_: 0.310*** Slope_H_: −0.330*
Test of joint significance of PPC variables [family management and family management^2^] (*p*-value)	0.000
Test of joint significance of all the variables (*p*-value)	0.0009
Test of joint significance of all variables in the model	0.006

**Figure 2:**
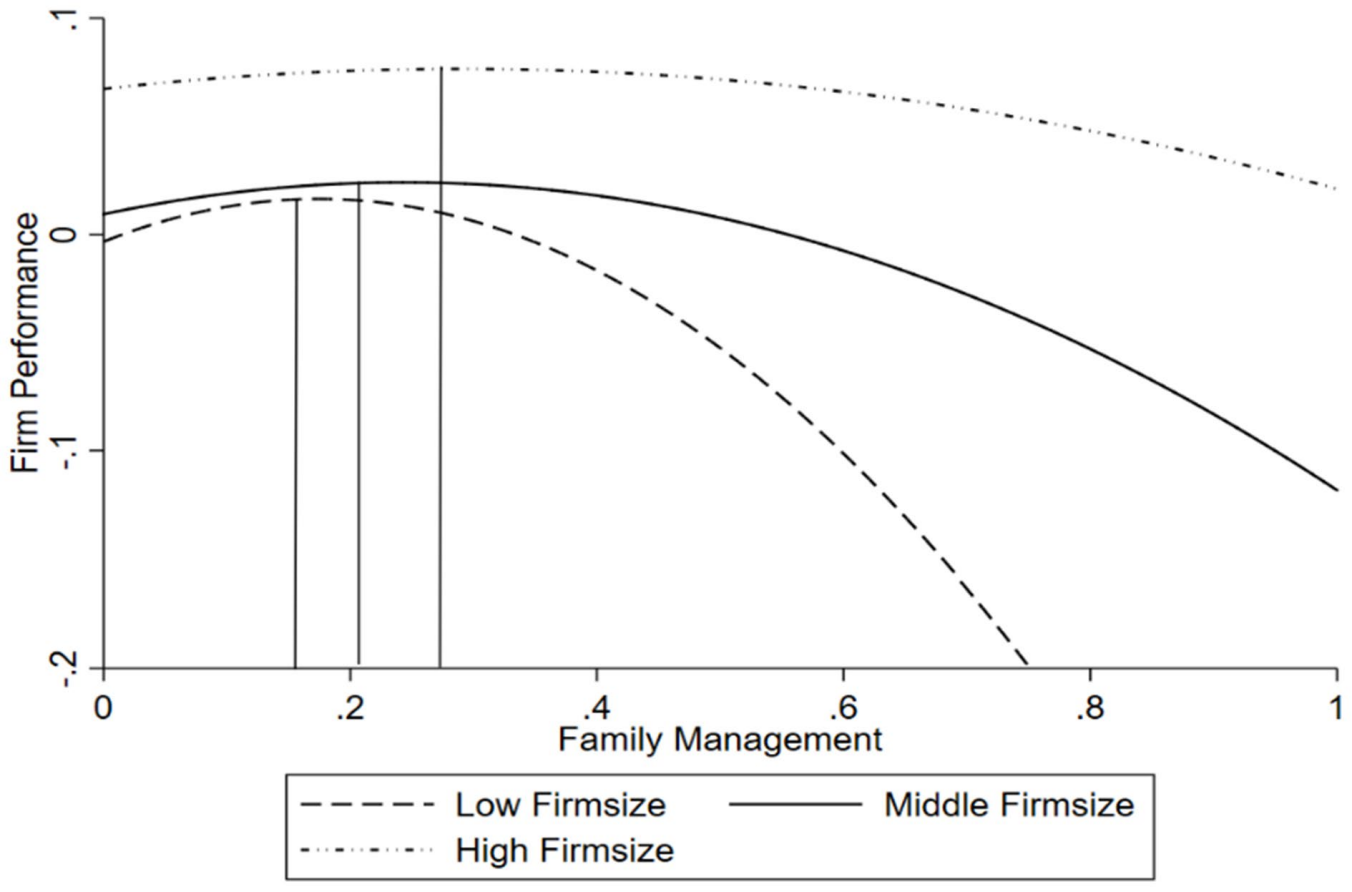
The modertating effect of firm size.

Table 3 also presents the results of the moderating effects of firm size and firm age (Model 3 and Model 4), with Model 5 as the full model. To test the moderating effect of firm size, Model 3 includes the interaction term between the family management and firm size (Family Management × Firm Size), as well as the interaction term between the squared term of the family management and firm size (Family Management^2^ × Firm Size). The results indicate that the coefficient for Family Management × Firm Size is significantly negative (β_3_ = −0.103, *p* < 0.05), and the coefficient for Family Management^2^ × Firm Size is significantly positive (β_4_ = 0.239, *p* < 0.05). These results remain significant in the full Model 5, indicating that the inverted U-shaped curve becomes flatter when firm size increases. The direction of the turning point’s movement depends on $\frac{\beta_{1}\beta_{4}-\beta_{2}\beta_{3}}{2{\left(\beta_{2}+\beta_{4}M\right)}^{2}}$ , and whether it is significantly different from 0 (see Haans et al., 2016 for a detailed derivation). Since the denominator of the equation is always greater than or equal to 0, the sign of the equation depends on the numerator, Specifically, when β_1_ × β_4_-β_2_ × β_3_ > 0, the turning point shifts to the right as the moderating variable M increases, and vice versa. Based on the coefficients in Model 3 in Table 3, β_1_ × β_4_-β_2_ × β_3_ is significantly positive, indicating that the turning point shifts to the right as firm size increases. To present more intuitive results, we plot the inverted U-shaped relationship for high, medium, and low values of firm size, as shown in Figure 2. Thus, hypothesis 2 is supported.

**Figure 3:**
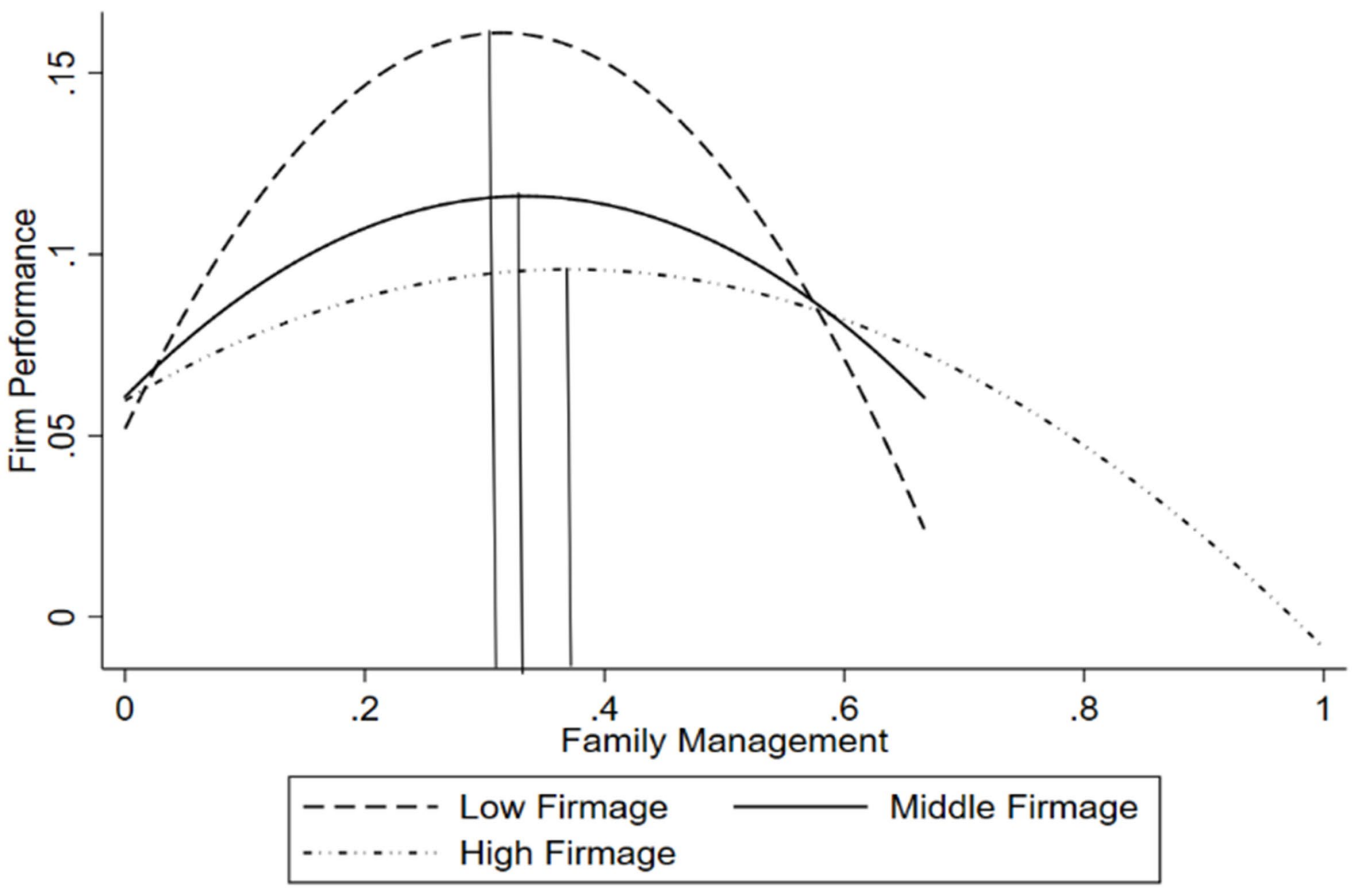
The modertating effect of firm age.

Similarly, Model 4 tests the moderating effect of firm age. Model 4 adds the interaction term between the family management and firm age (Family Management × Firm Age), and the interaction term between the squared term of the family management and firm age (Family Management^2^ × Firm Age). The results show that the coefficient of Family Management × Firm Age is significantly negative (β_3_ = −0.500, *p* < 0.001), and the coefficient of Family Management^2^ × Firm Age is significantly positive (β_4_ = 0.692, *p* < 0.1), indicating that the inverted U-shaped curve becomes flatter as firm age increases. Based on the coefficients of the variables in Model 4, the sign of β_1_ × β_4_-β_2_ × β_3_ is significantly positive, indicating that the turning point shifts to the right as firm age increases. To present more intuitive results, we plotted the inverted U-shaped relationship for high, medium, and low values of firm age, as shown in Figure 3. Thus, Hypothesis 3 is supported.

## 5 Robustness tests

We conducted several robustness tests to ensure the reliability of our results.

### 5.1 Robustness checks: alternative measure of non-economic goals

To capture the complex nature of non-economic goals, we conducted additional analysis using the proportion of family board members as an alternative measure of non-economic goals. The variable reflects the family’s control over residual claims, enabling families to maintain strategic control and prioritize non-economic goals over economic returns (Gomez-Mejia et al., 2007). While family management reflects the family’s direct involvement in daily operations and short-term decision-making authority, family board embodies the family’s legal control over the firm, ensuring long-term strategic autonomy. By employing family board as an additional measurement, we address the potential limitations of relying solely on family management and provide a more comprehensive understanding of the family’s non-economic intention (Chrisman et al., 2012). The results presented in Table 5, are consistent with our primary findings, further validating the robustness of our conclusions.

**Table 5:** Adding dependent variable: family board.

Variables	Model 1	Model 2	Model 3	Model 4
	Performance	Performance	Performance	Performance
Family Board	0.462^**^	0.377^*^	0.495^**^	0.414^**^
	[0.193]	[0.195]	[0.194]	[0.197]
Family Board^2^	−0.802^**^	−0.632^*^	−0.876^**^	−0.698^*^
	[0.377]	[0.383]	[0.380]	[0.387]
Family Board × Firm Size		−0.012		−0.022
		[0.057]		[0.057]
Family Board^2^ × Firm Size		0.873^**^		0.870^**^
		[0.353]		[0.355]
Family Board × Firm Age			−0.253	−0.269
			[0.166]	[0.167]
Family Board^2^ × Firm Age			2.238^**^	2.016^**^
			[0.958]	[0.962]
Firm Size	−0.008	−0.020^*^	−0.007	−0.019^*^
	[0.010]	[0.011]	[0.010]	[0.011]
Firm Age	0.008	0.007	−0.011	−0.008
	[0.025]	[0.025]	[0.027]	[0.027]
High Tech	0.012	0.008	0.012	0.008
	[0.022]	[0.022]	[0.022]	[0.022]
Independent	−0.199	−0.176	−0.215	−0.194
	[0.136]	[0.136]	[0.136]	[0.137]
Duality	−0.015	−0.015	−0.016	−0.015
	[0.014]	[0.014]	[0.014]	[0.014]
Succession	−0.011	−0.010	−0.010	−0.007
	[0.018]	[0.018]	[0.018]	[0.018]
Asset	−0.001	−0.001	−0.001	−0.001
	[0.004]	[0.004]	[0.004]	[0.004]
Beta	−0.024	−0.024	−0.022	−0.022
	[0.020]	[0.020]	[0.020]	[0.020]
Equity	0.104	0.102	0.094	0.092
	[0.063]	[0.063]	[0.063]	[0.063]
FM Age	0.000	0.000	0.000	0.000
	[0.002]	[0.002]	[0.002]	[0.002]
FM Edu	−0.001	−0.003	−0.002	−0.003
	[0.015]	[0.015]	[0.015]	[0.015]
Year	Yes	Yes	Yes	Yes
Industry	Yes	Yes	Yes	Yes
_cons	0.112	0.401	0.156	0.378
	[0.251]	[0.266]	[0.252]	[0.269]
adj. *R*^2^	0.183	0.184	0.185	0.186
*N*	2,877	2,877	2,877	2,877

*p < 0.1; **p < 0.05; and ***p < 0.01. Standard errors in brackets.

### 5.2 Endogeneity test

To address potential endogeneity concerns, we employ an instrumental variable approach. Following prior studies (e.g., Fang et al., 2022), we use the average size of the management team as our instrumental variable. This variable is calculated as the average number of managers for all firms within each industry, state, and year. The rationale for this choice is that the variable shapes the structural context in which firms operate. For example, larger average management teams may signal greater professionalization or resource availability within an industry or region, reducing the reliance on family managers, which is likely to influence the firm’s management structure (e.g., the proportion of family managers) but is unlikely to be directly correlated with firm performance or other firm-specific outcomes.

Following (Hamilton and Nickerson, 2003), we employed a two-stage regression approach with instrumental variables to address endogeneity. Table 6 reports the regression results of the endogeneity test. In the first stage, the instrumental and control variables were used to estimate the predicted value of family management, with family management as the dependent variable and the average size of the management team as the independent variable. The regression results show a significant negative correlation (*β* = −0.084, *p* < 0.01), indicating that the average size of the management team significantly affects the degree of family management, confirming the validity of the instrumental variable. In the second stage, the predicted value of the instrumental variable estimated in the first stage is included as an additional control variable in the regression, resulting in findings consistent with the main model.

**Table 6:** Robustness test: endogeneity tests.

Variables	First-stage	Second-stage	Second-stage	Second-stage	Second-stage
	Family management	Performance	Performance	Performance	Performance
Average TMTsize	−0.084***				
	[0.015]				
PredictAverage TMTsize		−0.934**	−0.888**	−0.848**	−0.812**
		[0.402]	[0.402]	[0.401]	[0.401]
Family Management		0.314^***^	0.274^***^	0.300^***^	0.257^**^
		[0.097]	[0.100]	[0.097]	[0.100]
Family Management^2^		−0.307^**^	−0.227	−0.292^**^	−0.201
		[0.141]	[0.152]	[0.141]	[0.153]
Family Management × Firm Size			−0.100^**^		−0.090^**^
			[0.042]		[0.042]
Family Management^2^ × Firm Size			0.240^**^		0.245^**^
			[0.108]		[0.111]
Family Management × Firm Age				−0.487^***^	−0.480^***^
				[0.122]	[0.123]
Family Management^2^ × Firm Age				0.686^*^	0.798^**^
				[0.394]	[0.407]
Firm Size	−0.000	−0.008	−0.019*	−0.005	−0.015
	[0.005]	[0.010]	[0.011]	[0.010]	[0.011]
Firm Age	0.024**	0.026	0.025	0.028	0.025
	[0.011]	[0.027]	[0.027]	[0.028]	[0.028]
High Tech	−0.002	0.008	0.009	0.004	0.005
	[0.010]	[0.022]	[0.022]	[0.022]	[0.022]
Independent	−0.064	−0.267*	−0.292**	−0.307**	−0.329**
	[0.060]	[0.137]	[0.138]	[0.137]	[0.138]
Duality	0.082***	0.044	0.040	0.038	0.034
	[0.006]	[0.035]	[0.035]	[0.035]	[0.035]
Family Board	0.452^***^	0.423^**^	0.406^**^	0.391^**^	0.378^**^
	[0.033]	[0.192]	[0.192]	[0.191]	[0.191]
Succession	0.019^**^	0.004	0.002	0.005	0.003
	[0.008]	[0.019]	[0.019]	[0.019]	[0.019]
Asset	−0.001	−0.001	−0.001	−0.001	−0.000
	[0.002]	[0.004]	[0.004]	[0.004]	[0.004]
Beta	0.009	−0.019	−0.021	−0.016	−0.019
	[0.009]	[0.020]	[0.020]	[0.020]	[0.020]
Equity Concentration	0.023	0.115^*^	0.124^*^	0.107^*^	0.115^*^
	[0.028]	[0.064]	[0.064]	[0.063]	[0.064]
FM Age	−0.003^***^	−0.002	−0.002	−0.002	−0.002
	[0.001]	[0.002]	[0.002]	[0.002]	[0.002]
FM Edu	−0.000	0.001	0.001	−0.001	−0.001
	[0.007]	[0.015]	[0.015]	[0.015]	[0.015]
Year	Yes	Yes	Yes	Yes	Yes
Industry	Yes	Yes	Yes	Yes	Yes
_cons	0.292^***^	0.261	0.502^*^	0.192	0.434
	[0.112]	[0.262]	[0.278]	[0.262]	[0.280]
adj. *R*^2^	0.065	0.187	0.188	0.192	0.193
*N*	2,877	2,877	2,877	2,877	2,877

*p < 0.1; **p < 0.05; and ***p < 0.01. Standard errors in brackets.

### 5.3 Alternative dependent variables

We use an alternative measure of the dependent variable as the robust test (see Table 7). Return on Equity (ROE) for year t + 1 is used as an alternative to the original measure of firm performance. Employing this approach, we also found results that were consistent with those of the main model.

**Table 7:** Robustness test: alternative measure of dependent variable.

Variables	Model 1	Model 2	Model 3	Model 4	Model 5
	Performance 2	Performance 2	Performance 2	Performance 2	Performance 2
Family Management		0.247^**^	0.256^**^	0.241^**^	0.251^**^
		[0.098]	[0.100]	[0.097]	[0.100]
Family Management^2^		−0.287^**^	−0.261^*^	−0.283^*^	−0.257^*^
		[0.146]	[0.149]	[0.146]	[0.149]
Family Management × Firm Size			−0.030		−0.027
			[0.060]		[0.060]
Family Management^2^ × Firm Size			0.998^***^		0.948^***^
			[0.364]		[0.365]
Family Management × Firm Age				−0.266^**^	−0.258^**^
				[0.127]	[0.129]
Family Management^2^ × Firm Age				0.235	0.195
				[0.412]	[0.417]
Firm Age	−0.035^***^	−0.035^***^	−0.050^***^	−0.033^***^	−0.048^***^
	[0.010]	[0.010]	[0.012]	[0.010]	[0.012]
Firm Size	0.018	0.016	0.015	0.022	0.021
	[0.026]	[0.026]	[0.026]	[0.027]	[0.028]
High Tech	0.004	0.003	0.003	0.001	0.002
	[0.023]	[0.023]	[0.023]	[0.023]	[0.023]
Independent	−0.306^**^	−0.319^**^	−0.294^**^	−0.343^**^	−0.319^**^
	[0.142]	[0.142]	[0.143]	[0.142]	[0.143]
Duality	−0.033^**^	−0.044^***^	−0.044^***^	−0.044^***^	−0.044^***^
	[0.015]	[0.015]	[0.016]	[0.015]	[0.016]
Succession	−0.024	−0.028	−0.027	−0.027	−0.026
	[0.018]	[0.018]	[0.019]	[0.018]	[0.019]
Asset	−0.004	−0.004	−0.004	−0.003	−0.004
	[0.004]	[0.004]	[0.004]	[0.004]	[0.004]
Beta	−0.010	−0.013	−0.011	−0.012	−0.009
	[0.020]	[0.020]	[0.021]	[0.020]	[0.021]
Equity	0.125^*^	0.130^**^	0.086	0.127^*^	0.083
	[0.065]	[0.065]	[0.066]	[0.065]	[0.066]
FM Age	0.000	0.000	0.001	0.000	0.001
	[0.002]	[0.002]	[0.002]	[0.002]	[0.002]
FM Edu	−0.020	−0.019	−0.019	−0.020	−0.020
	[0.016]	[0.016]	[0.016]	[0.016]	[0.016]
Year	Yes	Yes	Yes	Yes	Yes
Industry	Yes	Yes	Yes	Yes	Yes
_cons	0.680^***^	0.664^**^	0.947^***^	0.632^**^	0.899^***^
	[0.258]	[0.258]	[0.281]	[0.259]	[0.282]
adj. *R*^2^	0.451	0.452	0.443	0.453	0.444
*N*	2,877	2,877	2,877	2,877	2,877

*p < 0.1; **p < 0.05; and ***p < 0.01. Standard errors in brackets.

## 6 Additional analysis: the pandemic period (year 2020–2023)

The COVID-19 pandemic leads to a significant exogenous shock characterized by trade disruptions, resource shortages, and economic downturns, which would change the firm’s behavior and goal settings. To address this change, we conducted an additional analysis during the pandemic period. Using data from 2020 to 2023, we collected 778 observations to explore how family firms balanced economic and non-economic goals during this extraordinary period.

Table 8 presents the results of the period of 2020–2023. Specifically, we observed that an increase in the pursuit of non-economic goals was negatively correlated with economic goals during 2020–2023. This finding contrasts with the inverted U-shaped relationship observed during the pre-pandemic period (2009–2019), suggesting that the pandemic fundamentally altered the dynamics of family firm goal pursuit. We hypothesize that this shift is driven by two key factors: for the ability perspective, the economic uncertainty and market volatility brought by the pandemic demanded greater managerial expertise and strategic flexibility, potentially disadvantaging family managers who may have lacked the necessary professional skills to navigate this challenging environment. For the willingness perspective, in the VUCA environment of the COVID-19 pandemic, family managers may have prioritized the preservation of SEW even at the expense of short-term financial performance (Gomez-Mejia et al., 2024). Our analysis also revealed a moderating effect of firm size and firm age during the pandemic. Specifically, we found that the negative relationship between non-economic goals and economic goals weakened as firm size increased. As firms grow larger, they tend to develop more sophisticated organizational structures and professional management teams, which can dilute the influence of family managers and promote a more balanced approach to goal pursuit. Larger firms also possess greater resources and capabilities, enabling them to better adapt to changing market conditions and mitigate the risks associated with conservative decision-making driven by SEW considerations.

**Table 8:** Additional analysis: the pandemic period (year 2020–2023).

Variables	Year: 2020–2023				
	Model 1	Model 2	Model 3	Model 4	Model 5
	Performance	Performance	Performance	Performance	Performance
Family Management		−0.351^***^	−0.282^**^	−0.345^***^	−0.281^**^
		[0.130]	[0.132]	[0.131]	[0.133]
Family Management × Firm Size			0.214^**^		0.211^**^
			[0.086]		[0.087]
Family Management × Firm Age				0.450	0.176
				[0.697]	[0.703]
Firm Size	0.030	0.025	0.028	0.025	0.028
	[0.025]	[0.025]	[0.025]	[0.025]	[0.025]
Firm Age	0.428	0.348	0.262	0.341	0.261
	[0.650]	[0.647]	[0.645]	[0.647]	[0.645]
High Tech	−0.009	−0.026	−0.016	−0.028	−0.017
	[0.190]	[0.189]	[0.188]	[0.189]	[0.188]
Independent	−0.243	−0.241	−0.334	−0.251	−0.336
	[0.272]	[0.270]	[0.271]	[0.271]	[0.272]
Duality	−0.022	−0.001	−0.003	−0.003	−0.004
	[0.035]	[0.036]	[0.035]	[0.036]	[0.035]
Succession	0.014	0.020	0.010	0.018	0.009
	[0.038]	[0.038]	[0.038]	[0.038]	[0.038]
Asset	−0.090	−0.094	−0.092	−0.097	−0.093
	[0.123]	[0.122]	[0.121]	[0.122]	[0.122]
BETA	0.034	0.026	0.025	0.025	0.025
	[0.028]	[0.028]	[0.028]	[0.028]	[0.028]
Equity	0.007	−0.003	−0.004	−0.002	−0.003
	[0.053]	[0.053]	[0.052]	[0.053]	[0.053]
FM Age	2.256	1.045	1.182	0.973	1.151
	[4.304]	[4.302]	[4.282]	[4.306]	[4.288]
FM Edu	0.000	0.000	0.000	0.001	0.001
	[0.015]	[0.015]	[0.015]	[0.015]	[0.016]
Year	Yes	Yes	Yes	Yes	Yes
Industry	Yes	Yes	Yes	Yes	Yes
_cons	−10.716	−5.551	−5.865	−5.236	−5.736
	[16.962]	[16.973]	[16.892]	[16.989]	[16.915]
adj. *R*^2^	0.405	0.389	0.375	0.390	0.378
*N*	778	778	778	778	778

*p < 0.1; **p < 0.05; and ***p < 0.01. Standard errors in brackets.

## 7 Conclusion and discussion

### 7.1 Conclusion

Family managers possess both the willingness and ability to achieve diverse goals, a combination that significantly influences the behaviors and outcomes observed in family firms (De massis et al., 2014). Their strong identification with both the family and the firm leads to a high level of willingness advantage (Wielsma and Brunninge, 2019), thereby promoting the achievement of firm’s economic goals. However, the willingness advantage is constrained by the law of diminishing marginal returns: as the proportion of family managers increases, the willingness advantage diminishes. Additionally, family manages face an ability disadvantage. As the appointment of family managers increases, the limited family talent pool may no longer provide sufficiently capable managers. It also becomes difficult to dismiss less competent family managers (Mazzi, 2011). This discrepancy in quality between the internal family talent pool and the external labor market becomes more pronounced, highlighting ability deficiencies. These deficiencies hinder the improvement of the firm’s performance and the realization of its economic goals. The presence of these opposing forces leads to an inverted U-shaped relationship between family management and firm performance, which manifests as from symbiosis to competition. Changes in firm age and size can affect the willingness and ability of family managers, thereby influencing this inverted U-shaped curve. As the firm grows in size and age, its formal and informal norms become more established, and organizational routines stabilize. This stability can enhance the commitment of managers and mitigate their ability deficiencies, resulting in a rightward shift and a flattening of the inverted U-curve.

### 7.2 Discussion

The current findings offer theoretical implications in the following ways. First, by addressing goal complexity in family firms through the perspective of willingness and ability, we provide new insights into the dynamic relationship between economic and non-economic goals in Chinese family firms. Previous research has often assumed that family firms exhibit an “either-or” relationship in pursuing these goals, implying that increasing one may come at the expense of the other. However, our study reveals that these two types of goals can initially promote each other, evolving from “symbiosis” to “competition,” This indicates that family firms’ goal pursuit reflects a multi-dimensional interactive relationship.

Second, for cross-cultural implications, our study’s focus on Chinese family firms, where Confucian values profoundly shape the pursuit of both economic and non-economic goals, raises important questions regarding the generalizability of these findings to other cultural contexts. While Gomez-Mejia et al. (2024) highlight the SEW-intensive and SEW-sensitive nature of family firms in Latin America and the Caribbean (LAC), our findings reveal similar dynamics in China, where cultural values such as Confucian familism, loyalty and long-term orientation shape SEW preservation. By linking these insights to Chinese cultural patterns, we not only enrich the SEW discourse but also pave the way for cross-regional comparative research, and responding to Gomez-Mejia et al.’s (2024) call to extend SEW findings beyond LAC to regions like Africa and Asia, linking our analysis to Chinese cultural patterns and offering a globally relevant perspective.

Finally, our study advances the family business literature by clarifying the dynamics of goal balancing in family firms across stable and crisis contexts. By analyzing the 2009–2019 period, we establish a robust baseline for understanding how family firms balance economic and non-economic goals under the long-term stable conditions, revealing consistent strategic patterns. A supplementary analysis of the 2020–2023 pandemic period enriches this framework by showing how extreme exogenous shocks intensify goal-driven trade-offs, such as prioritizing family control over firm performance. This dual-context approach bridges normative and crisis-driven behaviors, providing a nuanced theoretical lens to explore the resilience and vulnerabilities of family firms in turbulent environments.

### 7.3 Limitations and future research

Although this study offers important insights, limitations exist. First, given that non-economic goals in family firms are a multi-dimensional concept, due to data availability and the limitations of current research progress, this paper only considers family management and family board as the representative of non-economic goals, which may be somewhat one-sided. In future research, we will attempt to examine other dimensions of non-economic goals.

Second, the research conclusion primarily come from a sample of listed companies. Thus, caution should be exercised when generalizing these findings to smaller family firms. Compared to small and medium-sized non-listed companies, listed companies are generally larger in scale and more aligned with modern corporate governance practices. Consequently, large listed companies differ in goal selection and balancing multiple goals relative to small and medium-sized enterprises. Thus, the goal pursuits and balancing strategies of numerous non-listed family firms in China, particularly small and medium-sized enterprises, warrant further investigation.

## Data Availability

Publicly available datasets were analyzed in this study. This data can be found at: https://data.csmar.com/.
